# A comparative study of patients’ satisfaction with different levels of hospitals in Beijing: why do patients prefer high-level hospitals?

**DOI:** 10.1186/s12913-020-05507-9

**Published:** 2020-07-10

**Authors:** Chengsen Cui, Xu Zuo, Yujia Wang, Haiyan Song, Jingyu Shi, Kai Meng

**Affiliations:** 1 School of Public Health, Capital Medical University, No.10 Xitoutiao, Youanmenwai Street, Fengtai District, Beijing, 100069 China; 2 Beijing Friendship Hospital, Capital Medical University, No. 95 Yongan Road, Xicheng District, Beijing, 100050 China

**Keywords:** Comparative study, Patients’ satisfaction, Integration of medical resources, Medical Alliance

## Abstract

**Background:**

To promote the integration of medical resources, Beijing has built medical alliances since 2012, but this reform has not changed the state of disordered medical treatment. Patients are still willing to go to high-level hospitals for medical treatment. What causes patients to prefer high-level hospitals? To explore the reasons behind this preference for high-level medical treatment among patients and to guide patients to make visits in an orderly manner, we conducted this study and compared patients’ satisfaction with hospitals of different levels in the context of medical resource integration.

**Methods:**

This study conducted a questionnaire survey among 1250 patients who were selected in 18 medical alliances in Beijing from October to December 2016. The study type is a comparative study based on cross-sectional data. Patient satisfaction was the main outcome, and descriptive analysis, chi-square tests, nonparametric tests and binary logistic regression analysis were used. The level of statistical significance was set at *p* < 0.05.

**Results:**

The overall satisfaction score of the medical alliances was 3.375, and the satisfaction scores of core hospitals and cooperative hospitals were 2.77 and 3.07, respectively. The overall patient satisfaction rate was 44.62%, and the satisfaction rates of core hospitals and cooperative hospitals were 34.34 and 50.43%, respectively. The type of hospital and understanding of medical alliance policy were the factors associated with patient satisfaction with the medical alliance.

**Conclusions:**

The patients’ satisfaction with cooperative hospitals was higher than their satisfaction with core hospitals. Although the patients were more satisfied with the service attitude of the cooperative hospitals, they still preferred core hospitals due to their higher expectations for their medical treatment and greater trust in their medical technology. It is necessary to explore the establishment of closed medical alliances under the unified management of human and financial resources to enable medical alliances to become a community of common interests and provide integrated medical services for patients.

## Background

The “Chinese model” of health care reform since 2009 has achieved remarkable effects in the past decade [1, 2]. The accessibility of medical services and the health indicators of residents have been greatly improved.

In China, hospitals and primary medical institutions are the main institutions for patients to seek medical treatment. Hospitals are divided into three levels according to their functions and tasks [3]. The first-level hospital provides basic medical care, prevention, rehabilitation, and health care services to a community. The second-level hospital is responsible for providing diagnosis and treatment of common and frequently occurring diseases to several communities, receiving referral patients from primary medical institutions and tertiary hospitals and undertaking teaching, training and scientific research tasks. The tertiary hospital is a regional medical institution that provides specialized medical services to several regions. These kinds of hospitals provide prevention, healthcare and rehabilitation services and undertake clinical teaching, training and scientific research tasks [4]. In addition, there are many community health service centers as primary medical institutions in cities [5]. Their main functions are the initial diagnosis of common diseases, health guidance for chronic diseases, disease screening, monitoring and management of high-risk groups, prevention of infectious diseases and control and health education [6].

In China, medical resources are unevenly distributed and are mainly concentrated in “big cities” and “high-level hospitals” [7]. In addition, the lack of medical insurance system constraints and other factors leads to the phenomenon of “disordered medical treatment” [8]. Disordered medical treatment means that the patient does not seek medical treatment according to the classification and functional positioning of the hospital but instead goes to a high-level hospital for any disease. This behavior has resulted in a larger number of patient visits in core hospitals than in cooperative hospitals. The Chinese government proposed the hierarchical diagnosis and treatment system in the new health care reform of 2009 for the first time in hopes that patients can be guided to seek medical treatment in a nonmandatory way. Patients whose disease cannot be diagnosed and treated by primary medical institutions will be referred to a higher-level hospital for treatment. When the patient goes into recovery, they will be referred to the primary care institutions for long-term treatment or rehabilitation [9–11]. The medical alliance was proposed for the first time in 2012, which is a powerful push for the implementation of hierarchical diagnosis and treatment systems, strengthens the vertical integration of medical resources at different levels. Medical alliances are dominated by the government. According to the extent of cooperation, medical alliances can be divided into closed medical alliances and loose medical alliances. In the closed medical alliance, the personnel and property of the cooperative hospital are uniformly managed by the core hospital, and they have coherent benefits. Loose medical alliances are mainly based on medical technology cooperation, and there are many institutional barriers. In China, the largest number of medical alliances are loose medical alliances [12]. In a medical alliance, the medical institutions with the highest medical technologies are regarded as core hospitals (generally tertiary hospitals), and a certain number of other medical institutions are cooperative hospitals (including second-level general hospitals, first-level general hospitals and community health service centers). The core hospitals are responsible for efforts such as the diagnosis and treatment of difficult diseases, doctors’ training, teaching, and research. The cooperative hospitals are responsible for several functions, including multiple diseases, common disease diagnosis and treatment, rehabilitation of referral patients and public health tasks. A series of measures have been implemented in medical alliances. Total prepaid medical insurance and other payment methods have been adopted by hospitals. Doctors do not need to apply for a change in practice location, and they do not need a record of practicing with other hospitals in the medical alliance if they need to go to cooperative hospitals to treat patients. The continuous electronic health records and medical records, information sharing and mutual recognition of inspection results are strengthened among hospitals [13]. According to the disease spectrum, key disease diagnosis and treatment needs, core hospitals will send their doctors to cooperative hospitals to promote the integration of medical resources in various ways, such as the joint construction of specialties, clinical teaching, medical technology teaching, teaching rounds and scientific research cooperation [14].

Beijing began to establish medical alliances in 2012. By March 2018, 58 medical alliances had been built with regional boundaries, including 55 core hospitals and 528 cooperative hospitals, covering all 16 districts in Beijing. With the construction of medical alliances in Beijing, the average length of stay in the core hospital has been shortened. The function of hospitals became clearer. Core hospitals strengthened medical technology and information communication and improved the technical level of cooperative hospital doctors through various training activities and regular visits by medical experts to primary medical institutions. Medical alliances provide a continuous health record between core hospitals and cooperative hospitals. Patients can directly make an appointment at the community health service center for expert outpatient service, and they can be referred quickly through the green channel if needed [15]. However, the situation of disordered medical treatment has not been greatly improved [16]. The data have shown that the number of outpatients and emergency visits in tertiary hospitals in Beijing in 2012 was 80.723 million and reached 99.774 million at the end of 2017, reflecting a 23.60% increase. The number in primary medical institutions was 36.268 million in 2012 and 37.01 million in 2017, an increase of only 3.12% [17]. Based on the above data, the policy has, to a certain extent, increased the ability of tertiary general hospitals to attract patients, so the crucial reason why patients prefer high-level hospitals for medical treatment should be discussed [18].

Previous research on medical alliances in Beijing has indicated that patients’ choice of medical treatment and willingness to first visit primary care institutions were partly affected by patient satisfaction [19–24]. Patient satisfaction is a kind of cognition and response produced by patients after comparing their expectations and actual perceptions of medical services; satisfaction is thus regarded as a subjective evaluation of patients [25]. According to customer satisfaction theory, the higher the customer satisfaction, the higher the customer loyalty is, and the more likely the customer will repeat the purchase and recommend it to others. In the medical field, this theory means that patients are more likely to choose the hospital with which they are more satisfied [26]. According to the above data (the number of outpatients and emergency visits in tertiary hospitals and primary medical institutions in Beijing), patients in Beijing prefer to visit core hospitals. Does this mean that compared with cooperative hospitals, core hospitals are more reassuring and satisfying to Chinese patients? To explore the reasons for the preference for high-level medical treatment among patients and to guide patients to make visits in an orderly manner, we conducted a cross-sectional survey in 16 districts of Beijing to compare the differences in patient satisfaction between the core hospitals and the cooperative hospitals within a medical alliance.

## Methods

### Survey respondents

This is a comparative study based on cross-sectional data, and 1250 patients who were selected in 18 medical alliances in Beijing from October to December 2016.

In the “Beijing Main Functional Area Plan” published by the Beijing government on September 17, 2012, the 16 districts in Beijing were divided into four different functional areas. The A and B districts were classified as the “capital functional core area.” The C, D, E, and F districts were classified as the “urban functional development area.” The G, H, I, J, and K districts were classified as the “new area of urban development,” and the L, M, N, O, and P districts were classified as the “ecological conservation development area.” Therefore, this classification is adopted for the current addresses of the respondents in the questionnaire.

### Interview and questionnaire

First, this study included a 48-person interview with doctors using the qualitative interview method to explore their attitudes toward medical alliances. We did not set the number of interviewees in advance. Instead, according to the principle of “information saturation” in qualitative research, we stopped the interview process after the 48th interview when we thought the collected materials were sufficient. Ten personal in-depth interviews and 7 focus groups with 5–6 persons in each group were conducted. We used the interview guidelines to conduct the interviews. We prepared three questions: 1. What is the current status of the medical alliance?; 2.What are the current problems of the medical alliance?; and 3. What are the results and effects of the medical alliance? These questions are related to patient satisfaction.

We used the thematic framework analysis method to sort out and summarize the collected text data and formed different dimensions and questions of the questionnaire [27]. After the first version of the questionnaire was formulated, we conducted two rounds of expert discussion, inviting doctors, hospital administrators and staff of health administration departments to repeatedly discuss and modify the questionnaire. The experts agreed that the final version of the questionnaire was effective, reliable and scientific and could be used for questionnaire surveys. Finally, we conducted a test of reliability and validity. The Cronbach coefficient was 0.67, the KMO value was 0.60, the sample met the reasonable requirements for the data structure, *p* < 0.001 passed Bartlett’s test of sphericity, and the cumulative variance interpretation rate value was 54.04%. In summary, this questionnaire has good reliability and validity [28, 29].

The questionnaire includes 24 questions across four parts. The first part includes the characteristics of the patients (including gender, age, household registration, length of time at residence, current address, chronic disease status, patient type, medical insurance type, average monthly medical expenses), the second part is the patients’ degree of understanding and method of the medical alliance policy. The third part is the degree of utilization of the medical alliance (including patients’ willingness and reasons for choosing cooperative hospitals or core hospitals for treatment and the construction effect of the medical alliance), and the fourth part is the patients’ satisfaction with the medical alliance.

### Survey methods

The study used a stratified random sampling method to collect patients from 18 core hospitals and 80 cooperative hospitals of 18 medical alliances in 16 districts of Beijing. Because the number of medical institutions and health personnel in district C and district D is much higher than in other districts, two medical alliances were randomly selected from district C and district D, and one medical alliance was randomly selected from other areas to ensure the representativeness of the survey subjects.

The inclusion criteria of this study were patients aged between 18 and 85 years who had received medical services in the medical alliance. The exclusion criteria in this study were patients who had no autonomous behavior ability or awareness. To improve the valid response rate of the questionnaire, we distributed the questionnaire through government channels of the Beijing Municipal Health Commission and obtained informed consent from the respondents. We distributed 540 questionnaires in core hospitals. A total of 457 valid questionnaires were collected, and the valid response rate was 84.63%. We distributed 900 questionnaires in cooperative hospitals. A total of 793 valid questionnaires were collected, and the valid response rate was 88.11%. In total, 1250 questionnaires were collected, and the valid response rate was 86.81%.

### Patient satisfaction calculation method

The calculation method used for the overall satisfaction score is very typical in the international literature. Patients’ satisfaction score = (number of very dissatisfied patients * 1 + number of quite dissatisfied patients * 2 + number of neither satisfied nor dissatisfied patients * 3 + number of quite satisfied patients * 4 + number of very satisfied patients * 5)/total number of participants in the evaluation [30–32].

### Statistical analysis

The data were double-entered using Epidata 3.1 software to establish a database, and SPSS 20.0 was used for statistical analysis. A nonparametric test was used for the age and the average monthly medical expenses. A chi-square test was used for the type of hospital, gender, household registration, length of time at residence, current address, patient type, medical insurance type, chronic disease status, level of understanding of policy and method of understanding. *P* < 0.05 was considered statistically significant.

In logistic regression analysis, this study took the patients’ satisfaction evaluation of the medical alliance services as the dependent variable and reduced the dimensionality of the ordered dependent variable as a binary variable (very dissatisfied, quite dissatisfied, and neither satisfied nor dissatisfied were classified as dissatisfied; quite satisfied and very satisfied were classified as satisfied) [25]. The independent variables included the type of hospitals the patients visited, the basic information of the patients (gender, age, household registration, current address, length of time at residence, chronic disease status, patient type, medical insurance type, and average monthly medical expenses), and the patients’ cognition of medical alliances (levels of understanding and policy) (Table 1). Model 1 included the hospital type. Then, we introduced the confounding factor of hospital type in the next two steps. Model 2 included the type of hospital and patients’ basic information (gender, age, household registration, current address, length of time at residence, chronic disease status, patient type, medical insurance type, and average monthly medical expenses), and Model 3 included the type of hospital, patients’ basic information, and patients’ cognition of medical alliances (level of understanding of policy and method of understanding).

**Table 1:** Variable assignment

Characteristics	Variable	Assignment
Patients’ satisfaction	Y	1 = Dissatisfied; 2 = Satisfied
Type of hospital	X1	0 = Core hospital; 1 = Cooperative hospital
Gender	X2	0 = Male; 1 = Female
Age (years)	X3	1 = 0–20; 2 = 21–40; 3 = 41–60; 4 = 61–80; 5 = 81–100
Household registration		
Urban area	X4	0 = No; 1 = Yes
Suburbs	X5	0 = No; 1 = Yes
Nonnative	X6	0 = No; 1 = Yes
Residence time	X7	1 = Within half a year; 2 = Half a year to 1 year; 3 = One to two years; 4 = More than 2 years
Current address		
Capital functional core area	X8	0 = No; 1 = Yes
Urban functional development area	X9	0 = No; 1 = Yes
New area of urban development	X10	0 = No; 1 = Yes
Ecological conservation development area	X11	0 = No; 1 = Yes
Nonnative	X12	0 = No; 1 = Yes
Chronic disease status	X13	0 = No chronic diseases; 1 = Any chronic diseases
Characteristics	Variable	Assignment
Patient type	X14	0 = Inpatient; 1 = Outpatient
Medical insurance type		
UEBMI	X15	0 = No; 1 = Yes
URBMI	X16	0 = No; 1 = Yes
NMI	X17	0 = No; 1 = Yes
NRCMS	X18	0 = No; 1 = Yes
CI	X19	0 = No; 1 = Yes
Out of pocket	X20	0 = No; 1 = Yes
Average monthly medical expenses (yuan)	X21	1 = Less than 300; 2 = 301–500; 3 = 501–800; 4 = 801–1000; 5 = More than 1001
Level of understanding of medical alliance policy	X22	1 = Very little understanding; 2 = Little understanding; 3 = General understanding; 4 = Some understanding; 5 = High level of understanding
Method of understanding		
Media reports	X23	0 = No; 1 = Yes
Community promotion	X24	0 = No; 1 = Yes
Hospital promotion	X25	0 = No; 1 = Yes
Recommendations from relatives and friends	X26	0 = No; 1 = Yes
Others	X27	0 = No; 1 = Yes

*UEBMI* Urban employee-based medical insurance, *URBMI* Urban resident-based medical insurance, *NMI* National medical insurance, *NRCMS* New rural cooperative medical scheme, *CI* Commercial insurance

## Results

### Characteristics among patients

A total of 1250 patients participated in the survey, including 731 females (58.48%), of which 474 (37.92%) were aged between 41 and 60. Nearly half of the patients were from an urban area of Beijing, with 588 patients (47.04%) and 409 patients (32.72%) living in the new area of urban development. Most respondents (88.80%) lived in the city for more than 2 years. The average monthly medical expenses of 452 patients were less than 300 yuan, accounting for the highest proportion (36.16%) of the sample. Almost half of all patients (46.50%) participated in Urban Employee Basic Medical Insurance. In terms of chronic disease status, the number of people suffering from hypertension was the highest (31.59%).

### Single-factor analysis of patient satisfaction

To explore the factors affecting patient satisfaction with medical alliances, all variables were included in the analysis as independent variables using chi-square tests and nonparametric tests. The results showed that the type of hospital, the type of patient, the level of understanding of medical alliance policy and the method of understanding were statistically significant (*P* < 0.05). These variables were associated with patient satisfaction (Table 2).

**Table 2:** Single-factor analysis of patient satisfaction

Characteristics	Options	Satisfied	Dissatisfied	χ^2^	*P*
		N (%)	N (%)		
Type of hospital	Core hospitals	136 (12.41)	260 (23.72)	26.48	< 0.001
	Cooperative hospitals	353 (32.21)	347 (31.66)		
	Total	489 (44.62)	607 (55.38)		
Gender	Male	192 (18.06)	226 (21.26)	0.50	0.48
	Female	282 (26.53)	363 (34.15)		
	Total	474 (44.59)	589 (55.41)		
Age (years)*	0–20	4 (0.37)	2 (0.18)	−1.34	0.18
	21–40	135 (12.43)	182 (16.76)		
	41–60	177 (16.30)	241 (22.19)		
	61–80	151 (13.90)	153 (14.09)		
	81–100	17 (1.57)	24 (2.21)		
	Total	484 (44.57)	602 (55.43)		
Household registration	Urban area	234 (21.71)	286 (26.53)	1.68	0.43
	Suburbs	200 (18.55)	242 (22.45)		
	Nonnative	45 (4.17)	71 (6.59)		
	Total	479 (44.43)	599 (55.57)		
Length of time at residence	Two years or less	19 (1.84)	37 (3.59)	3.01	0.10
	More than two years	446 (43.30)	528 (51.26)		
	Total	465 (45.14)	565 (54.85)		
Current address	Capital functional core area	51 (4.83)	46 (4.36)	3.61	0.46
	Urban functional development area	116 (10.98)	144 (13.64)		
	New area of urban development	164 (15.53)	202 (19.13)		
	Ecological conservation development area	138 (13.07)	188 (17.80)		
	Nonnative	4 (0.38)	3 (0.28)		
	Total	473 (44.79)	583 (55.21)		
Patient type	Inpatient	137 (12.95)	221 (20.89)	8.05	0.01
	Outpatient	332 (31.38)	368 (34.78)		
	Total	469 (44.33)	589 (55.67)		
Medical insurance type	UEBMI	218 (19.95)	276 (25.25)	3.96	0.56
	URBMI	122 (11.16)	150 (13.72)		
	NMI	35 (3.20)	40 (3.66)		
	NRCMS	98 (8.97)	100 (9.15)		
	CI	6 (0.55)	11 (1.01)		
	Out of pocket	13 (1.19)	24 (2.20)		
	Total	492 (45.01)	601 (54.99)		
Chronic disease status	Any chronic diseases	312 (28.73)	371 (34.16)	0.78	0.21
	No chronic diseases	173 (15.93)	230 (21.18)		
	Total	485 (44.66)	601 (55.34)		
Average monthly medical expenses *	Less than 300	176 (16.45)	216 (20.19)	−0.07	0.95
	301–500	108 (10.09)	145 (13.55)		
	501–800	74 (6.92)	89 (8.32)		
	801–1000	50 (4.67)	60 (5.61)		
	More than 1001	68 (6.36)	84 (7.85)		
	Total	476 (44.49)	594 (55.51)		
Level of understanding of policy	Do not understand	373 (35.02)	570 (53.52)	59.04	< 0.001
	Understand	93 (8.73)	29 (2.72)		
	Total	466 (43.75)	599 (56.25)		
Method of understanding	Media reports	88 (8.76)	143 (14.24)	11.43	0.02
	Community promotion	128 (12.75)	122 (12.15)		
	Hospital promotion	153 (15.24)	176 (17.53)		
	Recommendations from relatives and friends	35 (3.49)	46 (4.58)		
	Others	42 (4.18)	71 (7.07)		
	Total	446 (44.42)	558 (55.58)		

*UEBMI* Urban employee-based medical insurance, *URBMI* Urban resident-based medical insurance, *NMI* National medical insurance, *NRCMS* New rural cooperative medical scheme, *CI* Commercial insurance

### Comparison of satisfaction between core hospitals and cooperative hospitals

The overall satisfaction score of the medical alliances is 3.375, and the satisfaction scores of core hospitals and cooperative hospitals are 2.77 and 3.07, respectively. The overall patient satisfaction rate is 44.62%, and the satisfaction rates of core hospitals and cooperative hospitals are 34.34 and 50.43%, respectively. The evaluation of cooperative hospitals is better than that of core hospitals. A nonparametric test was conducted on the satisfaction scores of core hospitals and cooperative hospitals. According to the nonparametric test (*P* = 0.009), there was a difference in the satisfaction scores between the core hospitals and cooperative hospitals.

### Logistic analysis of factors associated with patient satisfaction

To further explore the impact of hospital type on patient satisfaction, all variables were included in a binary logistic regression analysis in three models. The results showed that the type of hospital is always the factor associated with patient satisfaction with the medical alliance. In Model 3, the level of understanding of medical alliance policy is a factor associated with satisfaction (Table 3).

**Table 3:** Logistic analysis of factors associated with patient satisfaction (OR, 95% CI; *n* = 1250)

Variables	Model 1	Model 2	Model 3
Type of hospital	1.945 (1.507–2.509)*	1.675 (1.238–2.268)*	1.609 (1.154–2.243)*
Cooperative hospitals			
Gender		0.849 (0.644–1.118)	0.810 (0.601–1.091)
Female			
Age		0.931 (0.775–1.119)	0.831 (0.678–1.019)
Household registration			
Suburbs		1.108 (0.788–1.558)	0.979 (0.676–1.417)
Nonnative		0.983 (0.588–1.644)	1.140 (0.648–2.006)
Length of time at residence		1.254 (0.925–1.700)	1.170 (0.854–1.603)
More than two years			
Current address			
Urban functional development area		0.880 (0.530–1.462)	0.669 (0.385–1.161)
New area of urban development		0.787 (0.461–1.344)	0.719 (0.403–1.283)
Ecological conservation development area		0.649 (0.373–1.128)	0.704 (0.386–1.284)
Nonnative		2.089 (0.333–13.126)	1.087 (0.162–7.286)
Chronic disease status		1.039 (0.741–1.455)	0.996 (0.689–1.441)
Any chronic diseases			
Patient type		1.298 (0.949–1.773)	1.103 (0.782–1.556)
Outpatient			
Medical insurance type			
URBMI		0.954 (0.681–1.337)	0.995 (0.690–1.435)
NMI		0.859 (0.499–1.479)	0.782 (0.424–1.440)
NRCMS		1.115 (0.751–1.655)	1.181 (0.769–1.813)
CI		0.645 (0.186–2.232)	0.590 (0.143–2.435)
Out of pocket		0.399 (0.149–1.069)	0.391 (0.118–1.293)
Average monthly medical expenses		1.044 (0.942–1.159)	0.987 (0.881–1.106)
More than 300 yuan			
Understanding level of policy			2.544 (1.993–3.247)*
Method of understanding			
Community promotion			1.418 (0.932–2.158)
Hospital promotion			1.174 (0.787–1.752)
Recommendations from relatives and friends			1.089 (0.576–2.056)
Others			1.545 (0.897–2.661)
Constants	0.523	−1.19	−2.022

*P < 0.05, *UEBMI* Urban employee-based medical insurance, *URBMI* Urban resident-based medical insurance, *NMI* National medical insurance, *NRCMS* New rural cooperative medical scheme, *CI* Commercial insurance

### Analysis of the reasons for patients’ choices

Among patients’ reasons for choosing cooperative hospitals for treatment, the top three are convenience (32.90%), the high proportion of medical insurance reimbursement (19.39%) and the short waiting time (11.83%). The main reasons why people think it is difficult to seek medical treatment in core hospitals are “long wait time for medical treatment”, “difficulty in finding reliable doctors” and “less time to communicate with doctors” at 23.69, 15.31 and 12.11%, respectively.

## Discussion

From the above research results, the hospital type and level of understanding of medical alliance policy are the factors associated with patients’ satisfaction with the medical alliance, and cooperative hospitals have higher satisfaction than core hospitals do. According to other studies in China, the study results of Beijing, Wuhan, Yunnan, and Jilin are consistent with the results of this article [33–38]. Furthermore, a large proportion of the results of international studies in developing countries and developed countries are also consistent with those presented in this article [39–44]. However, the difference is that more foreign patients go to primary medical institutions for medical treatment, while more Chinese patients go to core hospitals for medical treatment. Due to the large differences in health care systems in different countries, the reasons for this phenomenon are different.

The Chinese government’s financial investment in cooperative hospitals is lower than that in core hospitals, and the medical income of core hospitals far exceeds that of cooperative hospitals [45]. Core hospitals with funds, technology and personnel advantages should provide more satisfying services. However, the results of this study are contrary to study assumptions and public beliefs [46, 47]. What causes the results of the study, specifically, that patients’ satisfaction with cooperative hospitals in the medical alliance is higher than that with core hospitals? First, different functions and tasks are undertaken by the two kinds of hospitals. In the cooperative hospital, the treatment effects are better with less complex diseases, so the satisfaction is higher. Second, the service attitude of cooperative hospitals is relatively good. Doctors have more time to diagnose a disease and address the concerns of a patient. At the same time, the cooperative hospital has undertaken many public health tasks and maintained a good doctor-patient relationship with local patients [48]. Studies have shown that the “patient-oriented” new medical service model is more conducive to establishing a strong and intimate long-term relationship with patients, allowing patients to have good autonomy and satisfaction. Moreover, the medical technology of cooperative hospitals has been greatly improved because of the construction of medical alliances, which is also one of the reasons for patients’ higher satisfaction with cooperative hospitals [49].

In terms of the core hospitals, long waiting times for medical treatment and inpatient beds, difficulty scheduling appointments, and less time to communicate with doctors are the reasons for dissatisfaction. Poor medical experiences naturally lead to a decline in satisfaction. Moreover, the diseases undertaken by the core hospitals are far worse than those in the cooperative hospitals, the complexity of the disease treatment is higher, and the treatment effects are not always satisfactory. When the treatment results of the diseases fail to meet the expectations of the patients, the patients will have profoundly negative emotions that may lead to violence. Studies have shown that patients have a strong willingness to communicate with doctors, and the lack of communication will affect their evaluation of the hospital and even lead to disputes between doctors and patients [50]. Core hospitals should focus on improving medical services, strengthening patient-oriented communication between doctors and patients, simplifying the admission procedure and shortening the waiting time so that patients can have a better medical experience.

Although the above findings indicate that Chinese patients are more satisfied with cooperative hospitals than core hospitals, most Chinese patients are still willing to choose core hospitals for medical treatment, which contradicts customer satisfaction theory. This phenomenon is caused by a variety of factors, even after the publication of hierarchical diagnosis and treatment and medical alliance policy. The insufficient compensation of public hospitals in China has resulted in profit-driven competition among hospitals [51]. Under the background of resource allocation dominated by market forces, the overcrowded tertiary hospitals have enough reasons to ask the government for more subsidies to finance their facility improvements and improve service capabilities, which has led to the development of improved tertiary hospitals. Moreover, tertiary hospitals are more influential because they have higher administrative levels in China’s government administration system, so they can ask for more medical resources. As tertiary hospitals improve their capacity, they become more competitive, attract more patients, and justify further government subsidies and investment [45]. Other hospitals work arduously to attract patients and develop capacity, resulting in the inadequacy of the service capacity of primary medical institutions, which drives most patients to high-level hospitals, resulting in the waste of medical resources [52, 53]. In addition, there are no measures requiring patients to make the first visit to a primary care institution. Therefore, the higher medical expectations of Chinese patients lead to disordered medical treatment. Meanwhile, the leverage effect of medical insurance in various regions of China is not ideal. The proportion of medical insurance reimbursement in different levels of hospitals is not large enough to play a role in guiding hierarchical diagnosis and treatment [54]. Finally, the drug types of core hospitals and cooperative hospitals vary greatly. Since primary health care institutions implement a basic drug system, the types of drug are more comprehensive in core hospitals. Once the patient is referred to primary health care institutions, it is difficult to ensure the supply of a drug and the continuity of treatment [55].

In addition, the results show that the more patients know about medical alliance policies, the higher their satisfaction with medical alliance services will be. However, there are many problems at present, such as insufficient publicity, a single publicity channel and a limited form of publicity. The government plays an important guiding role in the publicity of medical alliances and the shaping of medical treatment concepts. Therefore, the publicity of primary consultation, hierarchical diagnosis, and two-way referral should be promoted from the perspective of the patient and the disease diagnosis and treatment so that patients can better understand the benefits of medical alliances and have improved satisfaction.

From an international perspective, some developing countries, such as India, Cuba, Russia, South Africa and Chile, are in a period of rapid social and economic development, with defects in the healthcare system that are similar to those in China [56–60]. The healthcare system does not have a compulsory primary consultation, and patients can seek medical treatment in high-level hospitals freely, which may also lead to the phenomenon of disordered medical treatment, resulting in the low efficiency of the health service system and higher national health accounts, even reducing patient satisfaction [61]. Therefore, the results of this study and the reform experience in China can provide a reference for such countries. Meanwhile, other developing countries should avoid the problems with healthcare reform similar to those observed in China. After the establishment of the medical alliance in China, core hospitals and cooperative hospitals compete for patients as much as possible. In addition, doctors in core hospitals do not have enough motivation to refer patients and help cooperative hospitals. The main reason is that loose medical alliances cannot mobilize the enthusiasm of hospitals and doctors, and there are no common benefits. Therefore, it is necessary to establish closed medical alliances to provide integrated medical services for patients. The closed medical alliance solves the external problems of property rights, organization, personnel, and medical insurance and realizes unified management within hospitals. Closed medical alliances are considered to be the best form of comprehensive benefits, which can reduce the medical technology gap of core hospitals and cooperative hospitals, relieve the pressure of core hospitals, and improve patient satisfaction [62].

### Limitations

First, patients should be included in the interview before the development of the questionnaire. Second, due to the concentration of medical resources in Beijing, the core hospitals in Beijing are not only serving local patients but also nationwide patients, and in some specialist hospitals, 70% of the patients are from other provinces. Thus, the investigation of satisfaction with Beijing medical alliances may magnify the findings or contradictions. However, the number of cooperative hospitals in this study was far more than that of core hospitals, and 87.36% of the 1250 respondents were Beijing patients, so the limitations of this study have little effect on the results. Finally, satisfaction is only one aspect of patient attitudes, and it does not fully reflect all the problems of the Chinese health care system. Further empirical research is needed on the factors influencing patients’ choice of medical treatment and how to guide patients to seek treatment at different levels.

## Conclusions

The patients’ satisfaction with cooperative hospitals was higher than their satisfaction with core hospitals. Although the patients are more satisfied with the service attitude of the cooperative hospitals, they still prefer core hospitals due to their higher expectations for their medical treatment and greater trust in their medical technology. Although the Chinese government has proposed medical alliances, most of the loose medical alliances are unable to change the state of competition between hospitals and cannot reverse the phenomenon of disordered medical treatment. Therefore, it is necessary to explore the establishment of closed medical alliances under the unified management of human and financial resources to promote medical alliances to create a community of common interests and provide integrated medical services for patients. In addition, the policy publicity efforts of medical alliances should be strengthened. Through these methods, patient satisfaction will be improved.

## Supplementary information

**Additional file 1.** Questionnaire for Medical Alliance Patients.

## Data Availability

The datasets generated and/or analyzed during the current study are available from the corresponding author on reasonable request. E-mail: mengkai@ccmu.edu.cn.

## Abbreviations

KMO: Kaiser-Meyer-Olkin
UEBMI: Urban employee-based medical insurance
URBMI: Urban resident-based medical insurance
NMI: National medical insurance
NRCMS: New rural cooperative medical scheme
CI: Commercial insurance
OR: Odd ratio
CI: Confidence interval
