# Supplementary material for: A comparative study of patients’ satisfaction with different levels of hospitals in Beijing: why do patients prefer high-level hospitals?
Source: BMC Health Serv Res. 2020 Jul 10;20:643. doi: 10.1186/s12913-020-05507-9 (PMC7350561; doi:10.1186/s12913-020-05507-9)
Supplement: Supplementary file 1 — Additional file 1. Questionnaire for Medical Alliance Patients. [file 12913_2020_5507_MOESM1_ESM.docx]

Hospital Name:___________________ No：___________________

**Questionnaire for Medical Alliance Patients**

Dear Sir/Madam,

Hello! We are currently conducting a questionnaire survey on the construction of medical alliance in Beijing, with the purpose of understanding your cognition, utilize and satisfaction with medical alliance, and providing scientific reference for further improving the construction of medical alliance in Beijing. This questionnaire is filled in anonymously, and the survey results are only used for research, not for other purposes. You only need to fill in the questionnaire according to your actual situation, which is very important for our research!

Thank you for your support and cooperation!

Beijing Municipal Commission

Oct.2016

_______________________________________________________________________

Please tick "○" or "√" if appropriate.

- - 1. **Gender**______ A.Male B.Female
    2. **Date of birth**_______________Year______________Month
    3. **Household registration address**

A. Urban area B. Suburbs C. Non-native

- - 1. **Current address_________________________________________**
    2. **Residence time in Beijing__________**

A. Within half a year B. 6 months to 1 year

C. 1 year to 2 years D. More than two years

- - 1. **Patient type__________**

A. Inpatient B. Outpatient

- - 1. **Medical insurance type_________**

A. UEBMI B. URBMI C. NMI D. NRCMS

E. Commercial insurance F. Out of pocket

- - 1. **What chronic diseases do you have that have been diagnosed by doctors?**

1. None B. Hypertension C. Diabetes D. Stroke

E. Coronary heart disease F. Others___________

- - 1. **What is your actual monthly expenditure on medical treatment on average? (Personal payment after deducting medical insurance reimbursement)**

A. Less than 300yuan B. 301-500yuan C. 501-800yuan

D. 801-1000yuan E. More than 1001

- - 1. **When you think the disease is mild, which medical institution do you choose?**

A. Tertiary hospitals B. Second-level hospitals

C. First-level hospital or community health service centers

D. Instead of going to the hospital, go to the pharmacy and buy medicine

- - 1. **When you think the disease is serious, which medical institution do you choose?**

A. Tertiary hospitals B. Second-level hospitals

C. First-level hospital or community health service centre

D. Instead of going to the hospital, go to the pharmacy and buy your own medicine

- - 1. **What aspects do you think the difficulty in seeing a doctor mainly reflects? (Limited to three items)**

A. Difficulty in finding a trusted doctor B. long waiting time to see doctors

C. long time to pay for medicine D. difficulty in waiting for a hospital bed

E. Long appointment time for examination F. Short communication time with doctors

G. complicated medical procedures H. long distance from the hospital

I. Others________________________

- - 1. **How much do you know about medical alliance policies?**

A. Do not understand very much B. Not very understanding

C. General understanding D. More understanding E. Very much understanding

- - 1. **How did you learn about the medical alliance policy?**

A. Media reports B. Community promotion C. Hospital promotion

D. Relatives and friends recommend E. Others__________________

- - 1. **Will changes in health insurance policies (such as reimbursement rates) affect your medical choices?**

A. No influence B. Not obvious influence C. Little influence

D. Relatively great influence E. Significant influence

- - 1. **Are you willing to go to the primary medical institutions in the permanent area for the first time?**

A. Very reluctant B. Little reluctant C. No attitude

D. More willing E. Very willing

- - 1. **Are you willing to go to a primary medical institution in the resident area for rehabilitation treatment?**

A. Very reluctant B. Little reluctant C. No attitude

D. More willing E. Very willing

- - 1. **The main reasons why you are willing to go to primary medical institutions within the permanent resident area for first diagnosis or rehabilitation (limited to three)**

A. Convenient, close to home, easy to take care of

B. Higher reimbursement rate and lower out-of-pocket expenses

C. The treatment environment is more suitable for rehabilitation or follow-up treatment

D. Want to be referred to a major hospital through a community hospital

E. Large hospitals can contact community hospitals or rehabilitation hospitals to ensure continuity of treatment

F. Short waiting time in primary health care facilities

G. More detailed and comprehensive consultations with doctors in primary medical institutions

H. Access to family doctor services in primary medical institutions

I. The four categories of chronic diseases enjoy advantages in the community policy

- - 1. **The main reasons why you are not willing to go to primary medical institutions within the permanent residence area for first diagnosis or rehabilitation (limited to three)**

A. The referral process wastes time. It is better to go directly to A big hospital to delay the illness

B. Distrust the medical conditions of community hospitals. The technical level of doctors

C. The variety of drugs is not complete

D. Fewer inspection items

E. Drugs cannot be reimbursed, and the reimbursement rate for medical services is small

F. Community hospitals do not have beds and cannot be admitted

G. Others__________________

- - 1. **Have you ever been served by a medical alliance in your resident area?**

A. Yes B. No

- - 1. **How satisfied are you with the medical alliance service?**

A. Very dissatisfied B. somewhat dissatisfied C. neither satisfied nor dissatisfied

D. somewhat satisfied E. very satisfied

- - 1. **What convenience does the establishment of medical alliance bring you to see a doctor? (Limited to three items)**

A. No experience

B. Make an appointment at a community hospital to see a specialist at a major hospital

C. Reduction in self-paid medical expenses

D. Referral examinations through community hospitals are prioritized

E. Sharing and mutual recognition of inspection results to reduce duplicate inspection

F. Information sharing

G. Others________________

- - 1. **Among the services provided by medical alliance, which ones do you think are more attractive (limited to three)?**

A. Specialists from major hospitals make house calls in the community

B. The basic level can make an appointment to the expert number of a big hospital

C. Large hospital examination equipment can be Shared in the community to achieve mutual recognition of examination results and reduce duplication of examinations

D. Be able to transfer from the community to the superior hospital for direct hospitalization, reducing waiting time

E. Check and test the green channel (reduce waiting time, exempt registration fee, etc.)

F. In the medical consortium, there are more kinds of drugs, which can also be used in large hospitals at the grassroots level

G. Large hospitals are transferred to the community without recalculating the starting line

H. There is no need to re-enter personal information for referral from the community to a large hospital, and the diagnosis and treatment can be maintained continuously

I. Others________________

- - 1. **What convenience do you hope the development of medical alliance can bring to you in the future? (Limited to three items)**

A. Make an appointment at A community hospital with A specialist at A major hospital

B. Priority can be given to large hospitals through community hospital referrals

C. Sharing and mutual recognition of inspection results to reduce duplicate inspection

D. Increase the variety of medicines dispensed in community hospitals

E. The historical information of medical treatment can be Shared within different hospitals within the medical consortium

F. Reduce personal medical expenses
